# Inducible nitric oxide synthase (iNOS) expression may predict distant metastasis in human melanoma

**DOI:** 10.1038/sj.bjc.6690256

**Published:** 1999-03

**Authors:** W Tschugguel, T Pustelnik, H Lass, M Mildner, W Weninger, C Schneeberger, B Jansen, E Tschachler, T Waldhör, J C Huber, H Pehamberger

**Affiliations:** Division of Gynaecological Endocrinology and Reproductive Medicine, Department of Gynaecology and Obstetrics, University of Vienna, Währinger Gürtel 18–20, Vienna, A-1090, Austria; Division of General Dermatology, University of Vienna, Währinger Gürtel 18–20, Vienna, A-1090, Austria; Division of Immunology, Allergy and Infectious Diseases, Department of Dermatology, University of Vienna, Währinger Gürtel 18–20, Vienna, A-1090, Austria; Department of Clinical Pharmacology;, University of Vienna, Währinger Gürtel 18–20, Vienna, A-1090, Austria; Institute for Tumour Biology and Cancer Research, University of Vienna, Währinger Gürtel 18–20, Vienna, A-1090, Austria

**Keywords:** nitric oxide, human melanoma, metastasis

## Abstract

Expression of inducible nitric oxide synthase (iNOS) and its cellular localization was investigated in subcutaneous or lymph node metastases of human melanoma. Immunohistochemistry revealed that iNOS expression was limited to melanoma cells. In samples of patients without distant metastases, the number of iNOS+ tumour cells/total tumour cells was 55% ± 17% (*n* = 12) compared with 9% ± 8% when distant metastases of lung, liver or brain occurred within an observation period of 3 years (*n* = 10) (*P* < 0.001). Western blotting confirmed the expression of iNOS protein in select cases. Notably, iNOS is expressed in regional melanoma metastases and its expression is inversely related to the tumour's metastatic potential. Thus, iNOS expression may have predictive value for the development of distant metastases of human melanoma. © 1999 Cancer Research Campaign


*British Journal of Cancer* (1999) **79**(9/10), 1609–1612
© 1999 Cancer Research Campaign
Article no. bjoc.1998.0256

### Inducible nitric oxide synthase (iNOS) expression may predict distant metastasis in human melanoma

**W Tschugguel[1], T Pustelnik[2], H Lass[1], M Mildner[3], W Weninger[3], C Schneeberger[1], B Jansen[2,4], E Tschachler[3], T Waldhör[5], JC Huber[1] and H Pehamberger[2]**

[1]Division of Gynaecological Endocrinology and Reproductive Medicine, Department of Gynaecology and Obstetrics; [2]Division of General Dermatology and [3]Division of Immunology, Allergy and Infectious Diseases, Department of Dermatology; [4]Department of Clinical Pharmacology; [5]Institute for Tumour Biology and Cancer Research, University of Vienna, School of Medicine, Währinger Gürtel 18–20, A-1090 Vienna, Austria

**Summary** Expression of inducible nitric oxide synthase (iNOS) and its cellular localization was investigated in subcutaneous or lymph node metastases of human melanoma. Immunohistochemistry revealed that iNOS expression was limited to melanoma cells. In samples of patients without distant metastases, the number of iNOS+ tumour cells/total tumour cells was 55% ± 17% ($n$ = 12) compared with 9% ± 8% when distant metastases of lung, liver or brain occurred within an observation period of 3 years ($n$ = 10) ($P$ < 0.001). Western blotting confirmed the expression of iNOS protein in select cases. Notably, iNOS is expressed in regional melanoma metastases and its expression is inversely related to the tumour's metastatic potential. Thus, iNOS expression may have predictive value for the development of distant metastases of human melanoma.

**Keywords:** nitric oxide; human melanoma; metastasis

Nitric oxide (NO), synthesized enzymatically by nitric oxide synthases (NOS), has been shown to regulate vascular tone, central and peripheral nervous system signal transmission, and cell-mediated cytotoxicity (Moncada et al, 1991). Based on calcium dependence, three different NOS isoforms have been identified in human tissues: endothelial NOS (eNOS), neuronal NOS (nNOS), both of which are calcium dependent, and inducible NOS (iNOS), which is calcium independent. iNOS is transcriptionally regulated by a variety of growth factors and inflammatory cytokines, such as tumour necrosis factor α (TNF-α), interleukin 1 (IL-1), γ-interferon (IFN-γ), and bacterial lipopolysaccharide (LPS) (Moncada et al, 1991; Nathan et al, 1994). The induction of iNOS results in the production of high levels of NO, which is associated with a cytotoxic reaction mainly against pathogens (Schmidt and Walter, 1994). The role of NO in tumour biology, however, is still poorly understood. In situ studies have revealed that NOS expression is present in the tumour cells of solid human tumours (Thomsen et al, 1994). It has also been shown to be expressed in stromal cells of human breast carcinomas (Thomsen et al, 1995). Moreover, iNOS expression was shown in cultured K-1735 murine melanoma cells after appropriate stimulation with a combination of cytokines (Dong et al, 1994). However, its presence in situ in human malignant melanoma has not been reported. In the present study, we addressed this issue and investigated expression and cellular localization of NOS isoforms in human subcutaneous and lymph node melanoma metastases. Data on iNOS expression in these tissues were then correlated with both the potential of tumours to form distant metastases and the patients' survival time.

*Received 10 February 1998*
*Accepted 13 July 1998*

*Correspondence to:* W Tschugguel

### MATERIALS AND METHODS

### Patients

Subcutaneous or lymph node melanoma metastases (stage II–IV according to the UICC criteria) were obtained from 22 patients after informed consent. The specimens were divided and one part was fixed in neutral-buffered formalin for histological diagnosis (H and E histology), the other portion was snap frozen and stored at –80°C for subsequent immunolabelling and Western blot analysis. Tumour-free axillary lymph nodes of breast cancer patients were used as additional controls. Twelve of these 22 patients did not develop distant metastases during an observation period of 3 years (group 1), whereas the remaining ten patients developed lung, liver or brain metastases within 3–14 months (group 2). Four patients of group 1 and three patients of group 2 were women, the other patients were men.

### Immunohistochemistry

Immunohistochemistry was carried out as previously described (Tschugguel et al, 1996). In brief, the following antibodies were used on 6-μm frozen serial sections: murine monoclonal antibodies against human eNOS (2.5 μg ml$^{-1}$) and iNOS (5 μg ml$^{-1}$) (both were from Transduction Laboratories, Lexington, KY, USA), a rabbit polyclonal antibody against rat brain nNOS synthetic peptide (1:4000 dilution) (Biomol, Plymouth, PA, USA), a monoclonal HMB45 antibody or a polyclonal S-100 antiserum for identification of melanoma cells (respective dilutions were 1:100 and 1:200) (Dako, Glostrup, Denmark), and a polyclonal von Willebrand factor antibody (1:200 dilution) (Bio Genex, San Ramon, CA, USA). For leucocyte detection, adjacent sections

**Table 1** Association between clinical follow-up data and percentage of iNOS+ cells per high-power field (HPF) at time of surgery. Statistical analysis is described in the Results section

| Patient | Age at time of biopsy | Tissue[a] | Distant metastases[b] | Survival time in months | %iNOS+ cells/HPF |
|---|---|---|---|---|---|
| Group 1 | | | | | |
| DE | 89 | s.c. | – | Alive | 30 |
| GJ | 78 | s.c. | – | Alive | 60 |
| AI | 64 | l.n. | – | Alive | 50 |
| BK | 59 | l.n. | – | Alive | 40 |
| HJ | 78 | l.n. | – | Alive | 80 |
| SF | 71 | s.c. | – | Alive | 40 |
| GH | 74 | s.c. | – | Alive | 45 |
| WK | 79 | l.n. | – | Alive | 70 |
| KA | 78 | l.n. | – | Alive | 60 |
| VE | 41 | l.n. | – | Alive | 40 |
| SH | 56 | l.n. | – | Alive | 85 |
| KJ | 47 | l.n. | – | Alive | 60 |
| Group 2 | | | | | |
| SR | 86 | s.c. | 6 | 8 | 0 |
| SK | 54 | s.c. | 12 | 13 | 0 |
| BF | 87 | l.n. | 11 | Alive | 5 |
| KG | 65 | l.n. | 10 | 18 | 5 |
| TJ | 72 | l.n. | 7 | Alive | 10 |
| ST | 58 | l.n. | 13 | 18 | 0 |
| RF | 57 | l.n. | 13 | Alive | 15 |
| WM | 49 | l.n. | 2 | Alive | 10 |
| AG | 58 | l.n. | 5 | 7 | 20 |
| PT | 87 | l.n. | 2 | 3 | 25 |

[a]Tissue: s.c., subcutaneous metastasis; l.n., lymph node metastasis. [b]Time in months until occurrence of distant metastases (–, no distant metastases)

were incubated with a monoclonal antibody against the human leucocyte marker CD45 (Bio Genex) at 1:40 dilution. Negative controls were applied for all tissue sections by replacement of primary antibodies with appropriately diluted isotype-specific irrelevant immunoglobulins.

For immunohistochemistry, the slides were examined in a blind fashion by four independent observers (WT, TP, HL and WW).

### Quantification of iNOS+ cells and statistical analysis

Quantitative studies were carried out on sections stained with the monoclonal iNOS antibody. The number of tumour cells recognized as positively stained by this antibody was counted in three random and consecutive high-power fields with a × 40 objective, and expressed as a percentage of the total number of tumour cells counted in the same area.

The mean and the standard deviation of the number of iNOS+ cells from patients within groups 1 and 2 were calculated. The Wilcoxon statistical test was used to test the differences in the number of iNOS+ cells between both groups. Moreover, differences in survival times within a clinical follow-up time of 3 years were tested using the Cox regression model. Differences were considered significant when $P < 0.05$.

### Western blot analysis

For the analysis of iNOS protein expression, samples of frozen lymph nodes (melanoma lymph node metastases of group 1 patients, $n = 2$, and group 2 patients, $n = 3$; controls from axilla, $n = 3$) were homogenized and then lysed in NP40 lysis buffer as described previously (Pammer et al, 1996). After quantification, proteins were electrophoresed and blotted with an anti-iNOS monoclonal antibody (1.25 μg $ml^{-1}$) (Transduction Laboratories) or an appropriately diluted isotype matched IgG2a control monoclonal antibody. This was followed by an HRP-conjugated sheep anti-mouse antibody (1:10 000). Specific reaction products were detected by chemoluminescence (ECL-kit, Amersham).

### RESULTS

Immunohistochemistry was performed on 25 tissue samples consisting of six subcutaneous and 16 lymph node metastases of malignant melanomas and three tumour-free axillary lymph nodes.

Melanoma cells were identified by staining against HMB45 or S-100 protein (data not shown).

The results of iNOS immunostaining and its association with the patients' outcome are summarized in Table 1. Immunostaining for iNOS was most pronounced in group 1 samples (subcutaneous or lymph node metastases of malignant melanoma, no distant metastases; $n = 12$) (Figure 1A), of which a respective H and E staining is presented (Figure 1B). In these samples, 55% ± 17% of the melanoma cells contained iNOS. The iNOS-containing tumour cells were found dispersely distributed throughout the tumour (not shown) or appeared to be a distinct population within solid tumour islands (Figure 1A). In contrast, cells positive for the human leucocyte antigen CD45 (Figure 1C) were restricted to the tumour stroma and appeared to be consistently iNOS negative. iNOS+ melanoma cells and unstained neighbouring melanoma cells did not differ in cell morphology. The intensity of staining within individual cells varied. Control sections with the IgG2a isotype control

 © Cancer Research Campaign 1999

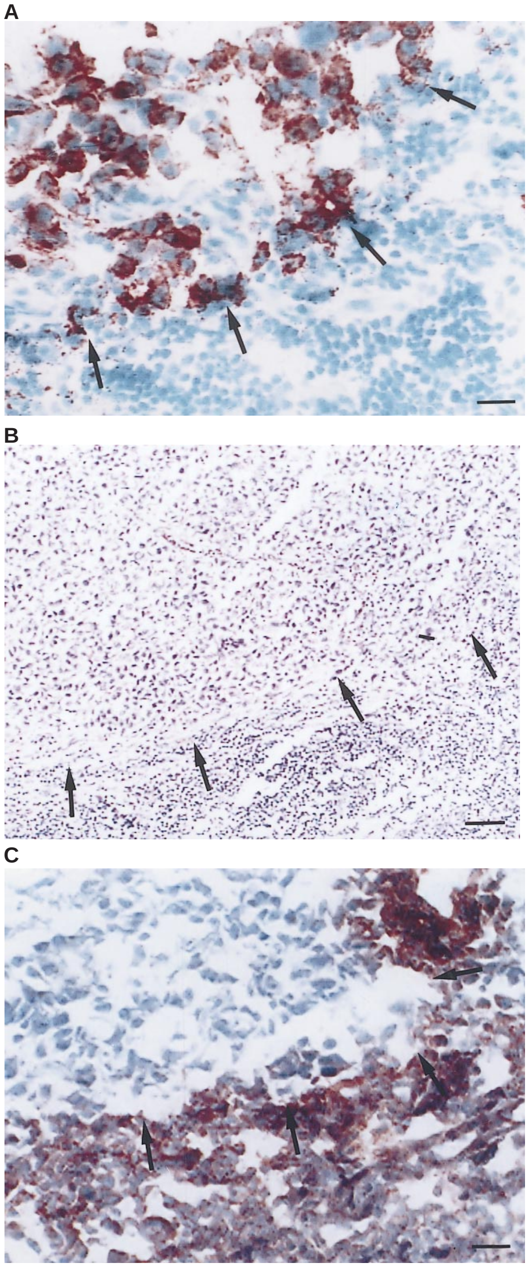

**Figure 1** Serial sections of a melanoma lymph node metastasis from patient ‘GJ’ of group 1 (subcutaneous or lymph node metastases of malignant melanoma, no distant metastases). (**A**) iNOS immunostaining is present within the majority of the tumour cells, which are localized among other unstained tumour cells (bar, 16 μm); (**B**) H and E histology (bar, 64 μm); (**C**) antigen localization with the CD45 antibody reveals that abundant CD45+ cells are localized within the stroma (bar, 16 μm); The arrows in (**A**), (**B**) and (**C**) indicate the border between tumour cells and stroma antibody showed no specific staining (data not shown). Group 2 samples (subcutaneous or lymph node metastases of malignant melanoma, formation of distant metastases within 2–13 months; $n$ = 10) had significantly fewer iNOS+ tumour cells (9% ± 8%) (Figure 2A) compared with group 1 samples ($P < 0.001$). Moreover, in group 2 samples, scarce CD45+ cells were intermingled between other unstained stroma cells (Figure 2B). Using the Cox regression model, no association was found between the

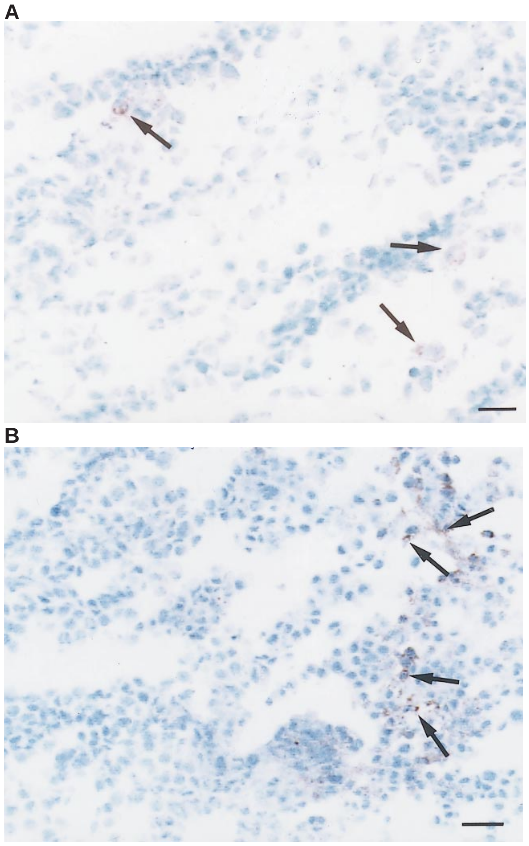

**Figure 2** Melanoma lymph node metastasis from patient ‘KG’ of group 2 (subcutaneous or lymph node metastases of malignant melanoma, formation of distant metastases within 3–14 months). (**A**) Note that only a few iNOS immunoreactive tumour cells (arrows) are intermingled between the majority of unstained tumour cells (bar, 16 μm); (**B**) antigen localization with the CD45 antibody reveals that scarce CD45+ cells (arrows) are localized within the stroma (bar, 16 μm)

number of iNOS+ tumour cells and cumulative survival ($P$ = 0.0707). In axillary lymph node samples ($n$ = 3), no iNOS staining could be detected (not shown). The specificity of the immunohistochemistry results was confirmed by Western blotting of select cases (Figure 3). We also excluded crossreactivity of the iNOS antibody with eNOS and nNOS antigens and demonstrated that eNOS staining was restricted to vascular endothelial cells (data not shown). eNOS staining furthermore corresponded to the distribution of von Willebrand factor. nNOS staining was not detectable in any of the lesions (data not shown).

### DISCUSSION

Our finding that iNOS expression may be negatively correlated with metastatic potential is in accordance with findings by Dong et al (1996), who demonstrated that non-metastatic K-1735 murine melanoma cells exhibited high levels of iNOS activity and NO release whereas metastatic cells did not. An inverse association between production of endogenous NO and the ability of K-1735 cells to survive in syngeneic mice to produce lung metastases was shown (Dong et al, 1996). Moreover, transfection of highly metastatic K-1735 cells, which express low levels of iNOS, with an iNOS expression vector rendered the cells non-metastatic by inducing high levels of NO production (Xie et al, 1995). These

*© Cancer Research Campaign 1999* 

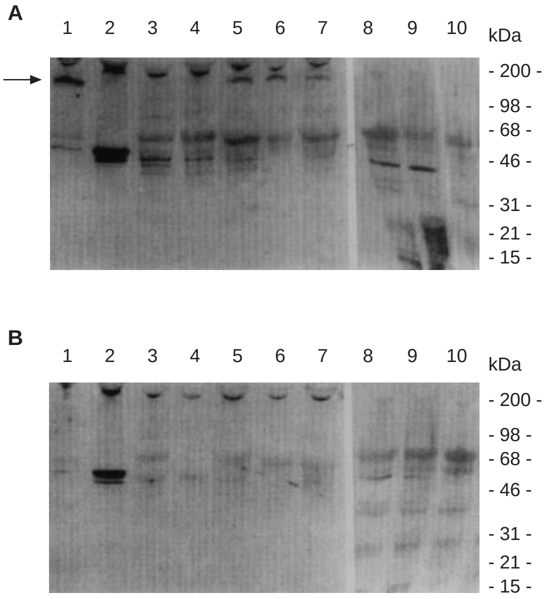

**Figure 3** Western blot analysis of iNOS expression (**A**) and respective negative controls with an isotype control antibody (IgG2a) (**B**) were performed on the following extracts: tumour-free axillary lymph nodes of two control patients (lanes 3 and 4) and malignant lymph nodes from patients 'HJ' (lane 5), 'GJ' (lane 6) and 'AI' (lane 7) of group 1, and of patients 'SR' (lane 8), 'SK' (lane 9) and 'ST' (lane 10) of group 2; a mouse macrophage lysate treated with IFN-γ and LPS for 12 h (transduction) was used as a positive control (lane 1) and an extract of human umbilical vein endothelial cells was used as a negative control (lane 2); an arrow indicates a 130-kDa specific band corresponding to iNOS in lanes 1, 5, 6 and 7 of **A**. Relative iNOS protein amounts were quantified by densitometric scanning and expressed by taking the IFN-γ and LPS-treated mouse macrophage lysate value as 1.0 (lane 1). This calculation revealed that the relative iNOS induction was 0.37-fold in lane 5, 0.23 in lane 6 and 0.16 in lane 7 compared with no such induction in lanes 8, 9 and 10

murine data as well as our results obtained in melanoma patients show an inverse correlation between iNOS expression and the ability of tumours to form distant metastases. Our findings suggest a significant inhibitory effect of tumour cell-derived NO on metastasis formation in human malignant melanomas.

Programmed cell death (apoptosis) is recognized as one of the main homeostatic mechanisms designed to block metastatic dissemination. Nitric oxide, a potent stimulus of apoptosis induction in K-1735 melanoma cells (Xie et al, 1996) has been reported to mediate apoptosis via expression of wild-type tumour-suppressor protein p53 (Meßmer et al, 1994). Accordingly, when the latter cells were transfected with mutant p53 cDNAs, they became metastatic, whereas parent and control-transfected cells remained non-metastatic (Koura et al, 1997). Additionally, nitric oxide-induced apoptosis in these cells is associated with down-regulation of Bcl-2, a proto-oncogene product that promotes cell survival and inhibition of apoptosis in various cells (Xie et al, 1997). In view of these considerations, we hypothesize that inducibly released nitric oxide may suppress the metastatic potential of human melanoma cells via induction of apoptosis. However, no significant correlation between the number of iNOS+ tumour cells and the survival time of patients could be seen. This may be caused by too short an observation time or our small sample size.

Notably, however, we could demonstrate that inducible nitric oxide synthase (iNOS) expression in human subcutaneous or lymph node melanoma metastasis seems to be inversely correlated with the tumour's ability to form distant metastases.

### ACKNOWLEDGEMENTS

We are grateful to Helga Herre and Ladislaus Szabó for excellent technical assistance and helpful methodical discussions. The study was supported in part by Schering Wien GesmbH., Vienna, Austria.

### REFERENCES

Dong Z, Staroselsky AH, Qi X, Xie K and Fidler IJ (1994) Inverse correlation between expression of inducible nitric oxide synthase activity and production of metastases in K-1735 murine melanoma cells. *Cancer Res* **54**: 789–793

Koura AN, VanGolen K, Tsan R, Radinsky R, Price JE and Ellis LM (1997) Regulation of genes associated with angiogenesis, growth, and metastasis by specific p53 point mutations in a murine melanoma cell line. *Oncol Rep* **4**: 475–479

Meßmer UK, Ankarcrona M, Nicotera P and Brüne B (1994) p53 expression in nitric oxide-induced apoptosis. *FEBS Lett* **355**: 23–26

Moncada S, Palmer RMJ and Higgs EA (1991) Nitric oxide: physiology, pathophysiology, and pharmacology. *Pharmacol Rev* **43**: 109–142

Nathan C and Xie QW (1994) Regulation of biosynthesis of nitric oxide. *J Biol Chem* **269**: 13725–13728

Schmidt HHHW and Walter U (1994) NO at work. *Cell* **78**: 919–925

Pammer J, Plettenberg A, Weninger W, Diller B, Mildner M, Uthman A, Issing W, Stürzl M and Tschachler E (1996) CD40 antigen is expressed by endothelial cells and tumor cells in Kaposi's sarcoma. *Am J Pathol* **148**: 1387–1396

Thomsen LL, Lawton FG, Knowles RG, Beesley JE, Riveros-Moreno V and Moncada S (1994) Nitric oxide synthase activity in human gynecological cancer. *Cancer Res* **54**: 1352–1354

Thomsen LL, Miles DW, Happerfield L, Bobrow LG, Knowles RG and Moncada S (1995) Nitric oxide synthase activity in human breast cancer. *Br J Cancer* **72**: 41–44

Tschugguel W, Knogler W, Czerwenka K, Mildner M, Weninger W, Zeillinger R and Huber JC (1996) Presence of endothelial calcium-dependent nitric oxide synthase in breast apocrine metaplasia. *Br J Cancer* **74**: 1423–1426

Xie K, Huang S, Dong Z, Juang SH, Gutman M, Xie QW, Nathan C and Fidler IJ (1995) Transfection with the inducible nitric oxide synthase gene suppresses tumorigenicity and abrogates metastases by K-1735 murine melanoma cells. *J Exp Med* **181**: 1333–1343

Xie K, Dong Z and Fidler IJ (1996) Activation of nitric oxide synthase gene for inhibition of cancer metastasis. *J Leukoc Biol* **59**: 797–803

Xie K, Wang YF, Huang SY, Xu L, Bielenberg D, Salas T, McConkey DJ, Jiang WD and Fidler IJ (1997) Nitric oxide mediated apoptosis of K 1735 melanoma cells is associated with downregulation of Bcl 2. *Oncogene* **15**: 771–779

 *© Cancer Research Campaign 1999*
